# Reduced inferior fronto-insular-thalamic activation during failed inhibition in young adults with combined ASD and ADHD compared to typically developing and pure disorder groups

**DOI:** 10.1038/s41398-023-02431-4

**Published:** 2023-04-22

**Authors:** Steve Lukito, Owen G. O’Daly, David J. Lythgoe, John Hodsoll, Stefanos Maltezos, Mark Pitts, Emily Simonoff, Katya Rubia

**Affiliations:** 1 Department of Child and Adolescent Psychiatry, King’s College London, Institute of Psychiatry, Psychology and Neuroscience, London, UK; 2 Department of Neuroimaging, King’s College London, Institute of Psychiatry, Psychology and Neuroscience, London, UK; 3 Department of Biostatistics and Health Informatics, King’s College London, Institute of Psychiatry, Psychology and Neuroscience, London, UK; 4 The Adult Attention-Deficit/Hyperactivity Disorder (ADHD) and Autism National Service, Behavioural and Developmental Psychiatry Clinical Academic Group, South London and Maudsley Foundation NHS Trust, London, UK

**Keywords:** Diagnostic markers, ADHD, Autism spectrum disorders

## Abstract

Autism spectrum disorder (ASD) often co-occurs with attention-deficit/hyperactivity disorder (ADHD) and people with these conditions have frontostriatal functional atypicality during motor inhibition. We compared the neural and neurocognitive correlates of motor inhibition and performance monitoring in young adult males with “pure” and combined presentations with age-and sex-matched typically developing controls, to explore shared or disorder-specific atypicality. Males aged 20–27 years with typical development (TD; *n* = 22), ASD (*n* = 21), combined diagnoses ASD + ADHD (*n* = 23), and ADHD (*n* = 25) were compared using a modified tracking fMRI stop-signal task that measures motor inhibition and performance monitoring while controlling for selective attention. In addition, they performed a behavioural go/no-go task outside the scanner. While groups did not differ behaviourally during successful stop trials, the ASD + ADHD group relative to other groups had underactivation in typical performance monitoring regions of bilateral anterior insula/inferior frontal gyrus, right posterior thalamus, and right middle temporal gyrus/hippocampus during failed inhibition, which was associated with increased stop-signal reaction time. In the behavioural go/no-go task, both ADHD groups, with and without ASD, had significantly lower motor inhibition performance compared to TD controls. In conclusion, only young adult males with ASD + ADHD had neurofunctional atypicality in brain regions associated with performance monitoring, while inhibition difficulties on go/no-go task performance was shared with ADHD. The suggests that young people with ASD + ADHD are most severely impaired during motor inhibition tasks compared to ASD and ADHD but do not reflect a combination of the difficulties associated with the pure disorders.

## Introduction

Approximately 28% of people with autism spectrum disorder (ASD) meet criteria for attention/deficit-hyperactivity disorder (ADHD) [1]. Population registers showed higher rates of co-occurring ADHD in young adults with ASD than any other age groups [2, 3], which could indicate diagnostic persistence over time. Symptom profiles, age of onset and distribution of ADHD diagnostic subtypes in the two groups are largely similar [4, 5]. However, the two conditions appear to differ neurocognitively; reduced cognitive control functions such as motor inhibition are more consistently observed in ADHD [6, 7], while lower cognitive control in ASD often are associated with the co-occurring ADHD symptoms [8–12], giving rise to the hypothesis of additive neurocognitive difficulties in the combined ASD + ADHD group relative to the “pure” groups [12, 13].

Such difficulties of cognitive control in ADHD and ASD, and their neural correlates, are frequently examined using the stop-signal task, which requires withdrawal of already triggered motor responses, and the go/no-go task, which requires selective withholding of prepotent responses [14]. Meta-analyses of cognitive control studies in ADHD, which predominantly include these two motor inhibition tasks, have shown underactivation in cognitive control and salience brain regions such as inferior frontal gyrus (IFG)/insula and striatum [14–17], with underactivation in right striatum and IFG, which are key regions of motor inhibition, found meta-analytically to be ADHD-specific relative to ASD [14].

Children and adults with ADHD also demonstrate neurofunctional underactivation relative to typically developing (TD) controls during error or performance monitoring, which is assessed through failed inhibition trials. Underactivation clusters were observed in dorsomedial/ anterior cingulate, left and right inferior and superior frontal regions [18–23], temporo-parietal ventral and dorsal attention network regions including precuneus and posterior cingulate [24], as well as in caudate and putamen [19, 20, 25].

In ASD, under- and overactivation have been found during cognitive control in frontal brain regions, with medial prefrontal underactivation being the most consistent meta-analytic finding [14, 26–28], as well as in posterior brain regions including left lingual gyrus, cerebellum and right inferior occipital cortex [14, 26]. Among studies investigating the motor inhibition tasks only, overactivation in left orbito- and dorsolateral prefrontal and underactivation in right dorsolateral prefrontal cortices, alongside clusters of over- and underactivation in posterior brain regions was ASD-specific when compared to ADHD. In addition, both ASD and ADHD shared underactivation in right anterior insula (AI) [14]. Finally, during error or performance monitoring, ASD children demonstrated increased medial frontal and left middle superior temporal activation compared to TD controls [29, 30].

Nevertheless, infrequently controlled co-occurrence of the conditions could have confounded the individual studies and meta-analyses findings in these disorders, which have motivated recent comparisons between “pure” ASD and ADHD groups. Two studies showed that during inhibition, ASD relative to ADHD children and TD controls showed increased right middle frontal gyrus (MFG) activation [31], and adolescent ADHD relative to ASD boys and TD controls showed specific underactivation in left orbitofrontal cortex and basal ganglia, whereas ASD-specific overactivation was found bilaterally in IFG [32], which highlight the most striking divergence in the atypical brain features in the two disorders, i.e. frontostriatal underactivation in ADHD and prefrontal overactivation patterns in ASD. During inhibition failures, medial/left MFG activation in ASD children increased with ADHD symptoms [33], suggesting a synergistic rather than simply additive atypicality, which resonated with a previous report of a relatively complex and severe pattern of atypical brain-behaviour association involving medial/lateral prefrontal regions in ASD + ADHD boys compared to the pure groups [34], during an impulsive-choice delay-discounting task.

A functional magnetic resonance imaging (fMRI) study of motor inhibition and performance monitoring in young adults with ASD and ADHD would be a useful addition to the literature, and elucidate the neural substrates of the high co-occurrence of the disorders in this age group [2, 3]. We therefore compared young adult males with ASD, ADHD, and both ASD + ADHD using a modified tracking fMRI stop-signal task that measures both motor inhibition and error/performance monitoring and a behavioural go/no-go task. In line with prior evidence, we hypothesised an underactivation in cognitive control and performance monitoring brain regions and impaired go/no-go task performance in ADHD relative to ASD and typically developing controls. Overactivation in cognitive control areas was expected in the ASD group, especially compared to ADHD, as previously shown by meta-analyses and direct comparison studies [14, 31, 32]. Finally, based on previous findings in ASD + ADHD boys [34] and the hypothesised additive impairment in the combined group relative to the pure disorder groups [12, 13], we expected the ASD + ADHD group to have a combined and possibly more severe pattern of neural atypicality and cognitive difficulties than observed in ASD or ADHD alone.

## Methods

### Participants

Participants were young adult males (*n* = 107) aged 20–27 years with ASD, ADHD and ASD + ADHD and typical development. All participants had full-scale IQ (FSIQ) ≥ 70 on the Wechsler Abbreviated Scale of Intelligence-2 [35]. Groups did not differ in handedness [36] and most (82%) were right-handed. Exclusion criteria were epilepsy, personality disorder, substance use disorder, lifetime history of bipolar disorder or schizophrenia or past head injury leading to loss of consciousness.

People with ASD and/or ADHD were recruited through adult neurodevelopmental clinics, support organisations, social media and an epidemiological cohort of ASD young adults (the Special Needs and Autism Project or SNAP [37]). Psychostimulants, withdrawn 48 h before the study, or selective serotonin reuptake inhibitors (SSRIs) were not exclusion criteria in the clinical groups. Participants completed a study comprising several fMRI tasks and a neurocognitive task battery [38]. Due to excessive motion (*n* = 3), poor response to the stop task (*n* = 12), or an incidental MRI finding (*n* = 1), only 91 participants were included in the final analyses.

The ASD (*n* = 21) group consisted of 18 participants with clinical diagnoses (seven autism; seven Asperger’s syndrome, four atypical autism) and three with consensus research diagnoses of ASD from a team of consultant psychiatrists and psychologists from SNAP (one autism; one atypical autism, one pervasive developmental disorder [PDD] unspecified), based on the International Classification of Diseases (ICD-10) [39]. No participants were prescribed psychotropic medications.

The ASD + ADHD (*n* = 23) group consisted of 19 clinically diagnosed participants (four autism, eleven Asperger’s syndrome, four atypical autism), and four with consensus research diagnoses of ASD (two atypical autism, two pervasive developmental disorder [PDD] unspecified) from consultant psychiatrists and psychologists in SNAP based on the ICD-10. Sixteen participants met the criteria for combined, and seven for inattentive presentation according to the DSM-5 [40] ADHD diagnostic criteria (nineteen received the diagnosis from consultant psychiatrists in specialist clinics, while four received research diagnoses from the SNAP team supported by parental interview from childhood). Five participants were taking psychostimulants (four methylphenidate [MPH], one dexamfetamine), one took SSRI (escitalopram) and one both medications (MPH, sertraline).

All participants with ADHD alone (*n* = 25) met the DSM-5 ADHD diagnostic criteria following assessments with consultant psychiatrists in specialist clinics. Fifteen participants met the criteria for combined, nine for inattentive and one for hyperactive presentation. Four participants were taking psychostimulants (three methylphenidate [MPH], one lisdexamfetamine), two SSRIs (sertraline, escitalopram) and one both medications (MPH, sertraline).

Typically developing (TD) controls (*n* = 22) were recruited locally, had no psychiatric diagnoses, were medication-free and scored below cut-off for ADHD and ASD traits on the Conners’ Adult ADHD Rating Scale (CAARS) [41] and the Social Responsiveness Scale-2 (SRS-2) [42] respectively. All participants gave written informed consent to volunteer; and were given travel reimbursement and £50 for participating. This study was in accordance with the Declaration of Helsinki and was approved by a local National Health Service Research Ethics Committee (13/LO/0373).

### Motor inhibition tasks

#### Modified fMRI stop-signal task

The modified visual tracking stop-signal task requires withdrawal of an already triggered motor response with the appearance of an unpredictable Stop signal, while simultaneously controlling for selective attention related to the oddball effect of the low-frequency Stop signals [43, 44]. This task consisted of 300 trials presented in a pseudorandomized sequence, completed in one block. In 66.7% of trials, participants responded to a left- or right-pointing arrow as fast as possible (Go, *n* = 200; 1000 ms duration), with an ISI jittered between 700 and 1000 ms. In 20% trials, interspersed in the stimulus sequence, a Go signal was followed by an upward arrow Stop signal (*n* = 60; 300 ms). The first Stop signal appeared 250 ms after a Go signal, and its onset delay was adjusted ±50 ms subsequently by a tracking algorithm (ranging from 50 to 900 ms), depending on the subjects’ probability of inhibition (PI), i.e. increasing if PI is <50% and decreasing if it is over 50%, making the inhibitory process equally challenging for everyone and ensuring the probability of successful inhibition reaches ~50% for each participant. Finally, to control for selective attention during the detection of rare Stop signal, in 13.3% trials Go arrows were presented diagonally upward to the left/right (Oddball Go, *n* = 40; 1000 ms duration and ISI jittered between 700 and 1000 ms), which were used as a contrast for responses to the Stop trials. The primary inhibitory task measure is the stop-signal response time (SSRT), computed using the integration method which subtracted the mean stop-signal delay from the nth fastest RT to Go (including responses to the Oddball Go and side-switched responses) ranked from the shortest to longest. The nth rank is determined by the multiplication of the probability of responding given the Stop signal with the total number of Go trials [45]. Only participants responding to >70% Go signal were included in the analyses to ensure a prepotent response tendency [18]. Secondary measures of executive performance include mean response time (MRT) to Go signals, intrasubject response time variability (RTV) to Go signals, and post-error response time slowing (PERTS), known as a typical behavioural adjustment after committing errors [24, 46, 47], which was calculated by subtracting MRT to Go after Successful Stop from MRT to Go after Failed Stop.

### Behavioural go/no-go task

The adult version of the MARS go/no-go task [48, 49] was completed outside scanner. It requires a button response to frequent Go trials (*n* = 220 trials), and response withholding to rare No-Go signals (*n* = 80 trials; 26.7%). Each trial begins with a 300 ms presentation of the Go (an airplane) or No-Go signal (an exploding bomb), followed by a fixed interstimulus interval of 700 ms. The task is split into two blocks with equal number of trials and trial types, to be completed once with each hand. The first block requires left finger responses toward left-facing Go signals, while the second block requires right finger responses toward right-facing Go signals. The key measure of motor inhibition for this task is the probability of inhibition, with secondary executive control response measures being the MRT and intrasubject RTV to Go signals, and the percentage of premature responses, which were defined as responses occurring between 200 ms pre-stimulus and 100 ms post-stimulus onsets, considered too late for the previous stimulus and too early for the present [49].

### Neuroimaging data acquisition and analysis

Imaging data were acquired on a General Electric MR750 3 T MR scanner (Chicago, IL) with an 8-channel head coil for signal reception at King’s College London, UK. A T1-weighted structural sagittal ADNI Go/2 ACC IR-SPGR structural scan was taken with inversion time/repetition time/echo time (TI/TR/TE) = 400 ms/7.312 ms/3.016 ms, flip angle = 11°, 196 slices, FOV = 27 cm × 27 cm, 256 × 256 matrix and slice thickness of 1.2 mm, while T2*-weighted echo planar images (EPI, 303 volumes) were taken sequentially, top to bottom, with TR/TE = 1800/27 ms, flip angle = 75°, FOV = 21 cm × 21 cm, 64 × 64 matrix, in-plane resolution = 3 mm, 40 slices, slice thickness/gap = 3 mm/0.3 mm. The EPI scans were whole-brain parallel to the inter-commissural plane.

Preprocessing a participant’s functional data was conducted using Statistical Parametric Mapping (SPM12) and included slice-time correction, realignment of EPI series to middle volume to correct head motion, co-registration with the individual’s structural T1 scan, segmentation, normalisation to the Montreal Neurological Institute (MNI) EPI template and smoothing with an 8 mm Gaussian kernel. Volumes with frame-to-frame motion >1 mm or mean global signal >1.5% standard deviation were linearly interpolated using values from neighbouring frames using ArtRepair toolbox of SPM12 [31] and participants’ data with >20% interpolated volumes were excluded from final analyses. Analyses were conducted separately at subject- and group-level to ease computational load. At the subject-level, event onsets, convolved with the canonical hemodynamic response function, were used to predict BOLD response, while covarying for six translational and rotational motion parameters to control for residual volume-to-volume head motion. A high-pass filter (128 s) was applied to reduce low frequency noise while a first-order autoregressive model corrected time series correlation.

Three contrasts of interest were used to investigate the neural correlates of (1) successful motor inhibition, i.e. Successful Stop—Oddball, (2) performance monitoring, i.e. Failed Stop—Oddball, both controlling for selective attentional processes, (3) selective attention, i.e. Correct Oddball— Go, with the Go trials modelled as implicit baseline. A conservative contrast (4) Failed Stop—Successful Stop was post-hoc investigated to model performance monitoring while controlling for motor inhibition [18, 22]. At the group level, within-group activations were analysed with uncorrected voxel at *p* < 0.001, and family-wise error (FWE)-corrected on the basis of cluster extent at *p* < 0.05. Whole-brain between-group analyses were conducted using univariate ANOVA with group as independent factor on SPM12. Average beta coefficients were extracted using the MarsBaR toolbox [50] from significant clusters, and from clusters from a threshold *p* value between 0.05 and 0.10, with family-wise error correction, if the clusters were within the regions that have been found to show atypical activation or underactivation among the clinical groups [32]. The extracted average beta coefficient underwent further post-hoc pairwise group comparisons, sensitivity analyses, and regression analyses as below.

### Statistical analysis plan

Behavioural and questionnaire data preparation and statistical analyses were conducted using the IBM SPSS Software 26 (Armonk, NY). Phenotypic measures were compared across groups using univariate ANOVAs for interval data and Chi-square statistics for nominal data. To investigate patterns of difficulties on the task performance measures, a series of univariate ANOVAs were used in our main analyses to compare group differences in the individual measures without covarying for IQ or medication status, since these variables are deemed intrinsically part of the group characteristics [51]. Post-hoc, we carried out pairwise group comparisons for all data were corrected with Tukey-Kramer method and, furthermore, sensitivity analyses while covarying for IQ, ADHD medication or any psychotropic medication, not to obtain better estimates rather to explore the robustness of group effects. Furthermore, characteristics of participant included into and excluded from the final analyses were explored using a series of univariate ANOVAs and *t-*tests (Supplement S2).

To elucidate further the nature of the group-differentiating brain activation clusters, we investigated the specificity of their association with task performance or disorder traits post-hoc in a series of multiple regression analyses (see models below). Each model used average Beta from each cluster as a dependent variable. In Model 1, brain activation was regressed on SSRT and PERTS as independent variables, while controlling for their diagnostic grouping by covarying for dummy variables ASD, ADHD and ASD + ADHD diagnosis with TD control as implicit baseline (for ease of interpretation of the regression coefficients, PERTS and SSRT were converted from milliseconds into seconds). In Model 2a, brain activation was regressed on ASD (SRS total score), ADHD traits (CAARS ADHD index), and the interaction term between these traits while again covarying the diagnosis groups. The interaction term was included given that error/performance monitoring tends to elicit neural underactivation in ADHD [19–22], and overactivation in ASD [29, 30]. Since the shared construct between diagnoses and traits may introduce collinearity among predictors, we repeated the latter analysis with the disorder trait predictors only as comparison (Model 2b). Each model was run with average Beta from four clusters as dependent variable. Thus, we used a corrected *p* value threshold of 0.0125 (i.e. alpha of 0.05 divided by four) for each model to determine significance.

Regression models:

$${(1) \,\mathrm{y}}\,=\,\mathrm{B}_{0} + \mathrm{B}_1{{\,}^{\ast}}{\mathrm{Group}}_{{\mathrm{ASD}},\, {\mathrm{ADHD}},\, {\mathrm{ASD}}+{\mathrm{ADHD}}}+\mathrm{B}_2{{\,}^{\ast}}{\mathrm{SSRT}} + \mathrm{B}_{3}{{\,}^{\ast}}{\mathrm{PERTS}}$$

$$\begin{array}{l}{(2\mathrm{a})\, \mathrm{y}}\,=\,\mathrm{B}_0 + \mathrm{B}_{1}{{\,}^{\ast}}{\mathrm{Group}}_{\mathrm{ASD},\,\mathrm{ADHD},\, {\mathrm{ASD}}+{\mathrm{ADHD}}}+\mathrm{B}_{2}{{\,}^{\ast}}{\mathrm{Trait}}_\mathrm{ASD}\\\qquad\qquad\,+\,\mathrm{B}_{3}{{\,}^{\ast}}{\mathrm{Trait}}_\mathrm{ADHD}+\mathrm{B}_{4}{{\,}^{\ast}}{\mathrm{Trait}}_\mathrm{ASD}{{\,}^{\ast}}{\mathrm{Trait}}_\mathrm{ADHD}\end{array}$$

$$(2\mathrm{b})\,\mathrm{y}=\mathrm{B}_{0} + \mathrm{B}_{1}{{\,}^{\ast}}{\mathrm{Trait}}_\mathrm{ASD}+\mathrm{B}_{2}{{\,}^{\ast}}{\mathrm{Trait}}_\mathrm{ADHD}+\mathrm{B}_{3}{{\,}^{\ast}}{\mathrm{Trait}}_\mathrm{ASD}{{\,}^{\ast}}{\mathrm{Trait}}_\mathrm{ADHD}$$

## Results

### Participant characteristics

Groups differed in FSIQ (*F*[3, 87] = 5.2, *p* = 0.002), which were higher in ADHD and TD relative to ASD (both *p*s ≤ 0.012), but not in age or handedness. Groups also differed in self-rated ADHD index (*F*[3, 86] = 30.6, *p* < 0.001) and SDQ hyperactivity domain (*F*[3, 86] = 62.9, *p* < 0.001), which was higher in ADHD and ASD + ADHD, than ASD and TD (*p*s < 0.001). Informant ratings were in line with self-rated ADHD symptoms in the clinical groups (Table 1). Finally, groups differed in autistic traits (*F*[3, 86] = 19.7, *p* < 0.001) with all clinical groups being higher than TD (*p*s < 0.001), although informant ratings showed that ASD + ADHD had higher autistic traits than the ADHD group (*p* < 0.001). Compared to those included in the analyses, excluded participants had lower IQ, and higher informant ratings of ADHD index and autistic traits, although did not differ in their self-ratings of ADHD and autistic traits (Supplement S2).

**Table 1:** Participant demographic characteristics.

	TD	ASD	ASD + ADHD	ADHD	Group comparison			
	(*n* = 22)	(*n* = 21)	(*n* = 23)	(*n* = 25)	*F*/*χ*^*2*^	*df*	*p*	*Post-hoc*
Age (SD), years	23.0 (1.3)	22.8 (0.9)	23.1 (1.3)	23.1 (2.0)	0.22	3, 87	0.89	--
FSIQ (SD)	118.5 (12.1)	102.0 (19.8)	109.2 (14.8)	116.0 (13.2)	5.2	3, 87	0.002	ADHD, TD > ASD*
Left-handed (%)	4 (18.2)	4 (19.0)	5 (21.7)	4 (16.0)	0.40	--	0.98	--
Current stimulant (%)	--	--	6 (26.1)	5 (20.0)	0.25	--	0.74	--
Current SSRI (%)	--	--	2 (8.7)	3 (12.0)	--	--	<0.99	--
CAARS (*t*-score)								
ADHD index, self-rated	42.1 (8.0)	46.8 (8.5)	58.3 (11.8)	65.2 (7.7)	30.6	3, 86	<0.001	ADHD, ASD + ADHD > ASD***; TD***
ADHD index, informant-rated	--	47.8 (7.3)	66.0 (10.3)	63.9 (10.6)	22.3	2, 64	<0.001	ADHD, ASD + ADHD > ASD***
SRS-2 (*t*-score)								
Total score, self-rated	47.6 (6.3)	61.8 (9.1)	65.0 (10.3)	62.2 (7.0)	19.7	3, 86	<0.001	ASD, ADHD, ASD + ADHD > TD***
Total score, informant-rated	--	63.4 (8.2)	69.4 (11.5)	57.1 (10.9)	8.70	2, 64	<0.001	ASD + ADHD > ADHD***
SDQ (raw score)								
Hyperactivity/inattention,self-rated	1.7 (1.4)	3.1 (2.0)	7.0 (1.9)	7.4 (1.5)	62.9	3, 86	<0.001	ASD + ADHD, ADHD > ASD***, TD***; ASD > TD*
Hyperactivity/inattention,informant-rated	--	3.0 (1.6)	7.2 (1.7)	7.6 (1.7)	50.6	2, 63	<0.001	ASD + ADHD, ADHD > ASD***

*FSIQ* full-scale IQ, *CAARS* Conners Adult ADHD Rating Scale, *SRS-2* Social Responsiveness Scale version 2, *SDQ* Strengths and Difficulties Questionnaires.

***p < 0.001; *p < 0 .05.

### Motor inhibition task performance

#### Modified stop fMRI task

Mean of probability of inhibition for the modified stop-task across participants was 50.2% (Range: 41.7–60%), suggesting that the tracking algorithm was successful. There were no differences in the percentage of correctly responded “Go” across groups (*F*[3, 87] = 0.12, *p* = 0.95, Table 2). ANOVAs showed no significant group effects on SSRT, of which means, however, were in the expected direction across groups, i.e. higher in ASD + ADHD (*M* = 179.3, SD = 97.1; Hedge’s *g* = 0.48, *p* = 0.25), ADHD (*M* = 173.0, SD = 63.8; *g* = 0.48, *p* = 0.33), and ASD (*M* = 135.4, SD = 105.4 *g* = 0.11, *p* = 0.97) relative to TD controls (*M* = 121.2, SD = 141.0). No significant group effects were observe on the secondary measures MRT to Go trials, intrasubject RTV and PERTS (*F*s[3, 87] ≤ 1.11, *p* ≥ 0.35, Table 2). The findings remained after covarying for IQ, ADHD medication or any psychotropic medications (i.e. including both SSRIs and stimulants). Excluded compared to included participants had lower correct Go response (particularly in the ASD group), higher SSRT, and lower PERTS overall (Supplement S2).

**Table 2:** Behavioural task performance.

	TD	ASD	ASD + ADHD	ADHD	Statistics	
	(*n* = 22)	(*n* = 21)	(*n* = 23)	(*n* = 25)	*F*(3,87)	*p*
*Modified stop-signal task*						
Correct Go (SD), %	87.3 (7.55)	87.0 (6.43)	86.2 (7.41)	86.4 (6.02)	0.12	0.95
SSRT (SD), ms	121.2 (141.0)	135.4 (105.4)	179.3 (97.1)	173.0 (63.8)	1.68	0.18
PERTS (SD), ms	1.0 (53.3)	11.4 (57.8)	18.4 (37.1)	15.5 (55.5)	0.49	0.69
MRT to Go (SD), ms	636.2 (134.8)	586.6 (108.5)	574.7 (102.9)	602.3 (127.8)	1.11	0.35
Intrasubject RTV (SD), ms	162.7 (46.3)	155.6 (54.9)	146.4 (44.8)	151.0 (43.5)	0.49	0.69
*Behavioural go/no-go task*						
Prob. of inhibition (SD), %	85.8 (11.8)	75.8 (19.0)	67.1 (19.1)	66.7 (14.5)	6.93	<0.001***^(a)^
Prem. responses (SD), %	0.6 (1.0)	1.2 (1.9)	3.5 (8.8)	2.3 (3.0)	1.63	0.19
MRT to Go (SD), ms	301.3 (41.1)	293.8 (34.4)	289.5 (31.4)	299.6 (25.3)	0.61	0.61
Intrasubject RTV (SD), ms	58.3 (17.0)	65.2 (21.6)	69.1 (28.8)	71.2 (19.2)	1.52	0.22

*MRT* mean response time, *PERTS* post-error response time slowing, *prem responses* premature responses, *prob of inhibition* probability of inhibition, *RTV* response time variability, *SD* standard deviation, *SSRT* stop-signal response time.

***p < 0.001, ^(a)^ post-hoc analyses: TD > ASD + ADHD***, ADHD***, applying Tukey-Kramer multiple comparison correction method.

### Behavioural go/no-go task

A group effect was found on probability of inhibition (*F*[3, 87] = 6.93, *p* < 0.001), which was significantly reduced in ASD + ADHD (*M* = 67.1, SD = 19.1; Hedge’s *g* = 0.97, *p* = 0.001) and in ADHD (*M* = 66.7, SD = 14.5; *g* = 1.32, *p* < 0.001), but not in ASD (*M* = 75.8, SD = 19.0; *g* = 0.53, *p* = 0.19) relative to TD controls (*M*_TD_ = 85.8, SD = 11.8), corrected for multiple testing using Tukey-Kramer method. These group differences remained after covarying for IQ, ADHD medication, or any psychotropic medication. No significant group effects were observed on the secondary performance indices (*F*s[3, 87] < 1.63, *p* > 0.19; Table 2). Excluded compared to included participants, particularly among those in the ASD + ADHD group, had higher MRT and intrasubject RTV (Supplement S2).

### Brain activation during the modified stop-signal task

#### Motion

Groups did not differ in total volume-to-volume head movement (*F*[3,87] = 1.92, *p* = 0.13) or number of corrected volumes (*F*[3,87] = 1.69, *p* = 0.18).

#### Successful stop—Oddball trials

No group differences were observed for the contrast successful Stop—Oddball trials. Within-subject group activation is shown in Supplement S3.

#### Failed stop—Oddball trials

**Fig. 1:**
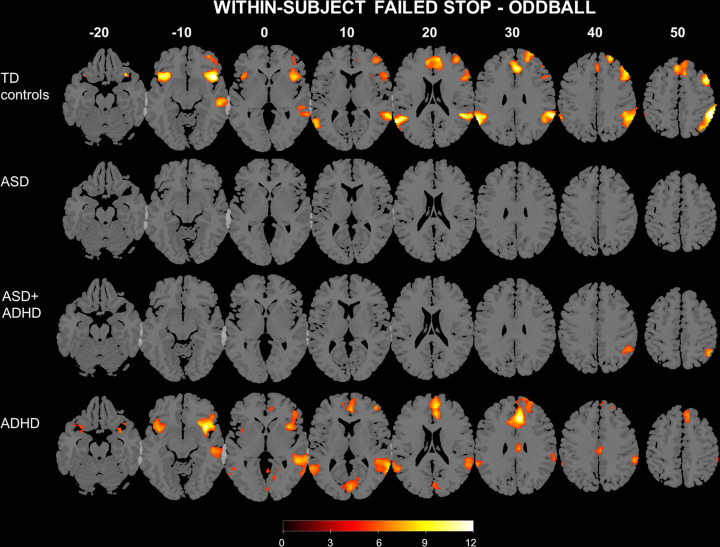
Whole-brain within-subject activation during Failed Stop contrasted with Oddball trials. Significant cluster of activation in groups of participants with typically development, ASD, ASD + ADHD and ADHD, formed with a peak voxel threshold of *p* < 0.001, uncorrected, and a cluster extent threshold of *p* < 0.05, applying family-wise error multiple comparison corrections.

Whole-brain within-group analyses showed that during failed Stop—Oddball trials (Fig. 1), TD controls activated right superior parietal lobe (SPL)/inferior parietal lobe (IPL)/supramarginal gyrus/angular gyrus (BA7/40/39) extending into right superior temporal gyrus (STG)/middle temporal gyrus (MTG) (BA22/21), right AI/IFG (BA13/47/44/46) and right MFG/dorsolateral prefrontal cortex (dlPFC)/superior frontal gyrus (SFG) (BA9/6), medial prefrontal cortex (mPFC)/dorsal anterior cingulate cortex (dACC) (BA24/32), left AI/IFG (BA13/47), and left IPL/supramarginal gyrus/angular gyrus/posterior STG/MTG (BA40/39/ 21/22). The ASD group showed no significant activation while the ASD + ADHD group activated right IPL (BA40). Last, the ADHD group activated right AI/IFG (BA13/47/45/46), left AI/IFG/MFG (BA13/47), mPFC/dACC (BA24/32), SFG (BA8/9/10), bilateral IPL/supramarginal gyrus/angular gyrus/ STG/MTG (BA40/39/21/22), ventral cingulate cortex (BA24) and right cuneus/cerebellum (BA17).

**Fig. 2:**
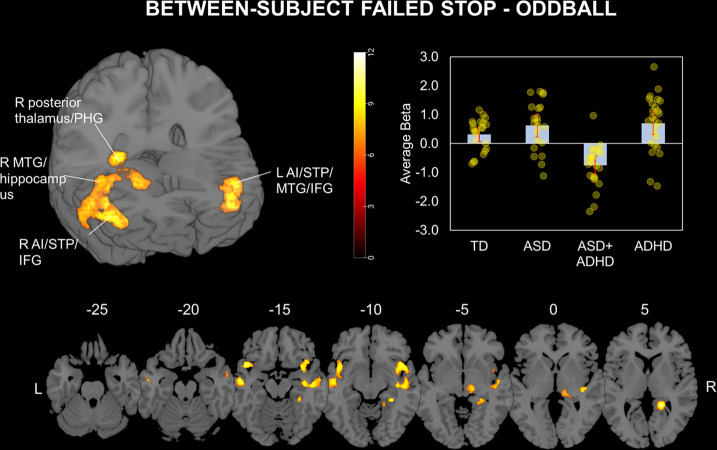
Whole-brain between-subject clusters of activation during Failed Stop relative to Oddball trials. Activation clusters were found during Failed Stop relative to Oddball trials in left anterior insula (AI)/superior temporal pole (STP)/middle temporal gyrus (MTG)/interior frontal gyrus (IFG); right posterior thalamus/parahippocampal gyrus (PHG); right MTG/hippocampus; and right AI/STP/IFG. Brain activation in the left AI/STP/MTG/IFG is presented in the bar chart across groups as an example, accompanied by error bars representing 95% confidence intervals in red and jittered scatterplots of individual activation in yellow. Activation clusters were formed using a peak voxel threshold of *p* < 0.001, uncorrected, and a cluster extent threshold of *p* < 0.05 family wise error-corrected for multiple comparisons.

Whole-brain between-group analyses (Fig. 2) revealed significant group effects in left AI/superior temporal pole (STP)/MTG/IFG orbital part (*p* = 0.014, *F* = 10.6, MNI peak coordinates [x = −44, y = 14, z = −12], cluster size[*k*_E_] = 346 voxels), right posterior thalamus/parahippocampal gyrus (PHG) (*p* = 0.013, *F* = 10.6, [18, −42, 6], *k*_E_ = 349) and in right MTG/hippocampus (*p* = 0.005, *F* = 9.39, [48, −16, −12], *k*_E_ = 434). A potentially weaker Group effect, although fell short of significance, was found in right AI/STP/IFG (*p* = 0.052, *F* = 9.43, [44, 12, −10], *k*_E_ = 237). However, since the region was situated the lateral frontostriatal area typically underactivated in ADHD, we still explored pairwise group differences of the extracted averaged beta coefficient of this cluster across groups. The post-hoc analyses indicated that ASD + ADHD had lower activation than the other groups in *all* four clusters (*p*s < 0.001 corrected with Tukey-Kramer method), which remained after co-varying for IQ, ADHD medication, or any psychotropic medication.

### Other imaging contrasts

No group effects were found for the contrasts Oddball – Go, and Failed Stop – Successful Stop trials. Within-subject clusters for the contrast Oddball – Go trials are reported in Supplement S3. No within-subject clusters were found for the contrast Failed Stop – Successful Stop trials.

### Analyses of brain-behavioural association

#### Association between brain activation and task performance indices

Multiple regressions showed selective association between brain activation with reduced SSRT in left AI/STP/MTG/orbital IFG (*B*_SSRT_ = −2.14, *p* = 0.010, 95% CI [−3.76, −0.52]), right posterior thalamus/PHG (*B*_SSRT_ = −1.70, *p* = 0.006, 95% CI [−2.90, −0.51) and in right AI/STP/IFG (*B*_SSRT_ = −2.36, *p* = 0.011, 95% CI [−4.17, −0.55]). The association in right MTG/hippocampus did not meet the corrected *p*-threshold of significance (*B*_SSRT_ = −1.38, *p* = 0.045, 95% CI [−2.73, −0.03]). No significant association was found between brain activation and PERTS (*B*_PERTS_ ≤ 0.58, *p*s ≥ 0.17), controlling for diagnostic grouping.

### Association between brain activation and dimensional ASD and ADHD traits

No significant associations were found between brain activation and ASD or ADHD traits, and their interactions, while covarying for diagnostic grouping in all four regions (*B*_trait ADHD_ ≤ 0.030, *p*s ≥ 0.48; *B*_trait ASD_ ≤ *−*0.020, *p*s ≥ 0.20; *B*_trait ADHD × trait ASD_ ≤ 0.043, *p*s ≥ 0.59). No significant associations were found between brain activation and those traits and their interactions in the model in which diagnostic grouping were not covaried, particularly in right posterior thalamus/PHG, right AI/STP/IFG and right MTG/hippocampus (*B*_trait ADHD_ ≤ 0.072, *p*s ≥ 0.133; *B*_trait ASD_ ≤ 0.016, *p*s ≥ 0.58; *B*_trait ADHD × trait ASD_ ≤ −0.070, *p*s ≥ 0.21). A potential weak association between activation in left AI/STP/MTG/orbital IFG and ADHD traits (*B*_trait ADHD_ = 0.099, *p* = 0.034) did not meet the corrected threshold of *p* = 0.0125. No significant association was found between left AI/STP/MTG/orbital IFG with ASD traits or interactions of traits (*B*_trait ASD_ = 0.040, *p* = 0.34; *B*_trait ADHD × trait ASD_ = −0.14, *p* = 0.066).

## Discussion

The main group comparison findings showed that young adult males with ASD + ADHD demonstrated underactivation in brain regions associated with performance monitoring, including primarily left AI/temporal cortex/orbital IFG and right AI/STP, right MTG/hippocampus and right posterior thalamus/parahippocampus relative to ASD, ADHD, and TD controls on the fMRI stop-signal task. Against our hypothesis, no finding of atypical brain activation clusters was found across groups during successful stop trials that indexes cognitive control. In the behavioural go/no-go task, reduced response inhibition was observed in the ADHD and the ASD + ADHD groups.

Performance monitoring, or error monitoring in typically developing adults, particularly implicates bilateral inferior frontal areas (i.e. AI/STP/IFG) [52–54], medial frontal, as well as midbrain and limbic regions including posterior thalamus, hippocampus, and parahippocampus [20, 55, 56]. The underactivation ventrolateral and medial prefrontal region [20, 22, 57], posterior thalamus, right hippocampus and parahippocampus [19, 25, 58] during performance monitoring have been shown in children and adults with ADHD. Based on these literature, the ASD + ADHD-specific underactivation found in this study may be described as ADHD-like. However, such interpretation in this study is constrained by the absence of similar underactivation in the ADHD alone group.

Performance monitoring during a stop-signal task consists of both conflict monitoring or withholding in the earlier Go process in case a Stop signal appears; and a later error processing to modify behaviour during failed stopping [54]. Behaviourally, no post-error slowing differences between groups or correlation between post-error slowing with brain activation were observed. We found instead a specific association between increased SSRT, i.e. poor motor inhibition, and decreased activation across clusters in left AI/STP/MTG/orbital IFG, right posterior thalamus/PHG and right AI/STP/IFG. This indicates that the performance monitoring clusters are associated with the earlier conflict monitoring related to motor inhibition problem instead of the later error processing [20, 59], which possibly reflect late-arriving motor inhibition that fails to intercede a triggered motor action [59, 60].

Interestingly, the brain activation was neither specifically associated with traits of ASD or ADHD, beyond the variation accounted already by diagnostic grouping or otherwise. A weak association between activation in one performance monitoring cluster in left AI/STP/MTG/orbital IFG with reduced ADHD traits was in the expected direction but did not meet the corrected threshold of significance. Taken together, the atypicality of brain activation tested in this manner appears more strongly associated with cognitive task performance, and less directly associated with the symptom severity of the diagnoses per se, which is in keeping with findings of the separability of cognitive difficulties from core diagnostic symptoms as shown in ADHD research [61].

In addition to the brain activation finding, difficulties of response withholding were also observed during the go/no-go task in the ASD + ADHD and in ADHD groups, with large effect sizes, relative to TD controls. While not significant statistically, both ADHD groups also demonstrated a pattern of increased mean SSRT relative to TD controls. Together these findings suggest that motor inhibition difficulties are primarily associated with ADHD diagnosis, which is in line with evidence from several behavioural studies showing increased difficulties of cognitive control or motor inhibition problems in ASD + ADHD relative to ASD alone [9, 10, 62, 63], and of specific associations between executive control and ADHD symptoms among individuals with ASD [11, 64].

The non-significant group differences in SSRT and the shared response withholding difficulties primarily in ADHD and ASD + ADHD during the go/no-go task, suggest a similarity between the two groups. However, the added presence of right IFG/AI and midbrain/limbic underactivation during error monitoring in ASD + ADHD suggested more severe neurofunctional atypicality in the combined group overall. A similar conclusion has been drawn previously in ASD + ADHD children and adolescents during an fMRI delay-discounting task [34]. Notably, both studies revealed patterns of specific neural atypicality in the ASD + ADHD relative to their age-matched ASD and ADHD controls, which suggests that individuals with ASD + ADHD do not simply have additive characteristics of the pure groups [12, 13].

The altogether absence of the hypothesised functional underactivation clusters in ADHD, while unexpected, is in line with a number of fMRI inhibition studies in ADHD adults [21, 22, 65–67]. Given the predictability of the fMRI task version over behavioural versions, the lack of observed underactivation could reflect heterogeneous activation patterns associated with the idiosyncratic strategies developed by the participants during the task that was not captured by the fMRI analysis [24, 68]. Alternatively, since there is far more evidence for inhibitory brain function underactivation in ADHD individuals with younger age [14, 69, 70], its absence in our young adult ADHD group could reflect neurofunctional maturation of the motor inhibition network. This speculative interpretation would require confirmation with direct comparison with younger participant groups or a longitudinal study design. Lastly, it is plausible that there is an altogether underestimation of group effects in brain activation related to the exclusion of individuals with higher severity of ASD or ADHD traits as judged from the informant ratings.

This study was constrained by some methodological limitations, including its relatively small sample size despite, to our knowledge, being the largest comparative fMRI study of motor inhibition in ASD and ADHD to date and the only one to compare the ASD + ADHD with the pure groups in young adulthood [31, 32]. The underactivation clusters found in the ASD + ADHD relative to other groups remains a neural correlate rather than a predictor for the condition. fMRI findings in relatively small sample sizes often showed low degree of reliability [71, 72]. Therefore, future studies with replication datasets in larger sample of the population is necessary, preferably incorporating prediction analysis for better reliability [73].

The increased sample homogeneity afforded by including young adult males only, selected due to the high prevalence of ASD and ADHD among males [74, 75], was at the cost of the generalisability of findings for the ASD and ADHD populations. Full-scale IQ distribution was unequal across groups, but was in the direction expected from the literature, i.e. lower among both groups with ASD diagnosis, presumably because a substantial proportion of individuals with ASD have lower IQ as found in population-representative cohorts [76, 77]. Our main group difference findings were presented without covarying for IQ [31, 51]. Subsequent analyses covarying for IQ was not completed to obtain adjusted group estimates of the main finding, but rather as a post-hoc sensitivity analysis to explore the robustness of group differences. Some strengths include the use of both fMRI and neurocognitive tasks, to provide a fuller picture of the cognitive profile across diagnostic groups, and the inclusion of well-characterised clinical groups from both clinical and population-representative samples, especially some individuals in the ASD and ASD + ADHD groups who have been followed longitudinally. Furthermore, there was a substantial proportion of medication-free individuals in the clinical groups.

To conclude, this study shows that young adult males with ASD + ADHD, but not those with ADHD alone, had underactivation in inferior fronto-insular-thalamic regions, reflecting earlier processes of inhibitory withholding during performance monitoring. On behavioural task, both ADHD groups with and without ASD were impaired in selective motor action withholding. Together the findings suggest that among young adults, those with ASD + ADHD have the most severe cognitive and neurofunctional atypicality, which do not appear to be a combination of the pure disorder forms.

## Supplementary information

Online supplementary materials
