# Supplementary material for: Reduced inferior fronto-insular-thalamic activation during failed inhibition in young adults with combined ASD and ADHD compared to typically developing and pure disorder groups
Source: Transl Psychiatry. 2023 Apr 22;13:133. doi: 10.1038/s41398-023-02431-4 (PMC10122665; doi:10.1038/s41398-023-02431-4)
Supplement: Supplementary file 1 — Online supplementary materials [file 41398_2023_2431_MOESM1_ESM.docx]

Supplementary materials for Lukito et al. (2023). **Reduced inferior fronto-insular-thalamic activation during failed inhibition in young adults with combined ASD and ADHD compared to typically developing and pure disorder groups. *Translational Psychiatry.***

| Supplementary S1. The modified fMRI stop-signal task description |
| --- |
| 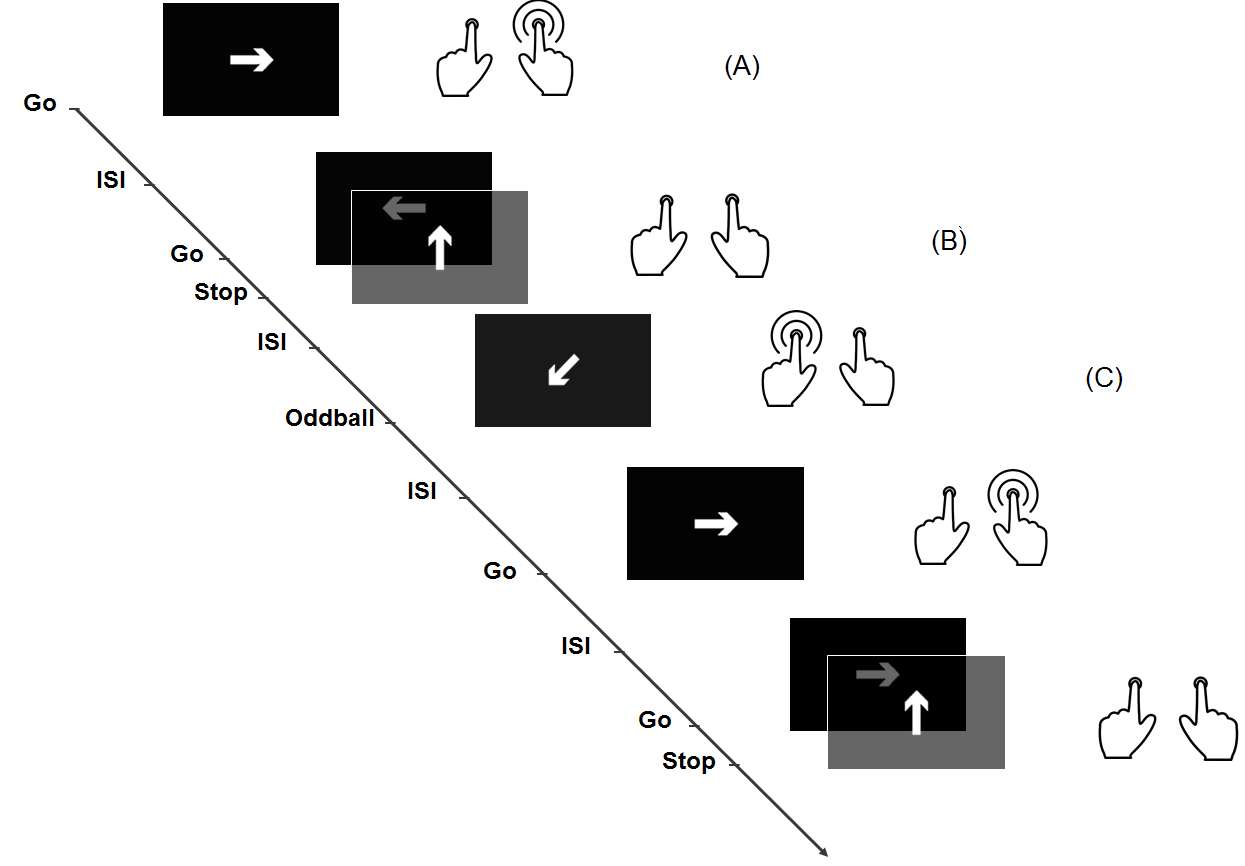 |
| Fig. S1. Modified fMRI stop-signal task. All stimuli were presented against a black background. The Go trials (A) consisted of arrows pointing horizontally to the left or right to be responded by left or right button press respectively. The Stop trials (B) consisted of a Go signal followed by a Stop signal, presented here on transparent background for clarity, arriving between 50 and 900ms after the Go signal, started from 250ms in the first trial. Participants were instructed to cancel their initiated response as best as they can in these trials. The Oddball trials (C) were arrows pointing diagonallly downward to the left or right and participants were instructed to respond by pressing the left or right button, respectively. Each trial was followed by an ISI jittered between 700-1000ms. The blue and red coloured arrow on the left indicated the time line of the events. Abbreviations. Go = Go cue, ISI = interstimulus interval, Stop = Stop signal, and Oddball = Oddball Go cue. |

**Supplementary S2. Characteristics of included and excluded participants**

Differences between included and excluded participants were assessed using a 2 (inclusions status: included, excluded) × 4 (diagnostic group: TD, ASD, ASD+ADHD and ADHD) ANOVA. Significant main effects of inclusion status were found for FSIQ (*F*[1,107]=7.79, *p*=.006), and informant-rated ADHD traits on CAARS, (*F*[1,107]=6.34, *p*=.014), and ASD traits on SRS2, (*F*[1,107]= 4.41, *p*=.039), with the findings showing that those excluded from compared to those included into the analysis had lower FSIQ, and higher ADHD and autistic traits (Table S3a). Significant interactions between inclusion status × group were found for age (*F*[3,107]= 3.67, *p*= 0.15), driven by exclusion of older participants in the TD group (*t*[24] = -3.83, *p*=.001); and for self-rated autistic trait (*F*[3,107]= 3.93 , *p*=.011), driven by exclusion of people with higher ASD traits in the ASD+ADHD group (*t*[25] = -2.58, *p*=.016).

During the modified stop-signal fMRI task, significant main effects of inclusion status were found for correct Go response (*F*[1,107]=118.4, *p*<.0005) and SSRT (*F*[1,107]=4.83 , *p*=.030), and PERTS (*F*[1,107]=9.12 , *p*=.003) (See Table S3b). A significant interaction between inclusion status × group was also found for correct Go response (*F*[3,107]= 3.74, *p*=.014), driven by exclusion of people with lower correct Go response in the ASD group (*t*[25] = -2.58, *p*=.016). Thus, those excluded compared to those included into the analyses had lower correct Go response (particularly in the ASD group), higher SSRT, i.e., lower motor inhibition control, and lower PERTS, i.e., an increase rather than decrease of speed following an error. During the go/no-go out-of-scanner task, significant main effects of inclusion status were found for MRT to Go (*F*[1,107]= 8.26, *p*=.005) and intrasubject RTV (*F*[1,107]=22.7, *p*<.0005). Significant interactions between inclusion status × group were also observed for MRT to Go (*F*[3,107]= 3.30, *p*=.023) and for intrasubject RTV (*F*[3,107]= 5.11, *p*=.003), in both cases driven by the exclusion of individuals with higher MRT to go and intrasubject RTV in the ASD+ADHD group (MRT to Go: *t*[25] = -3.09, *p*=.005; intrasubject RTV: *t*[25] = -4.04, *p*<.0005). Therefore, those excluded compared to included had later and more intrasubject response variability, particularly in the ASD+ADHD group.

**Table S2a. Characteristics of included and excluded participants**

|  | INCLUDED | | | | EXCLUDED | | | | Statistics | | | |
| --- | --- | --- | --- | --- | --- | --- | --- | --- | --- | --- | --- | --- |
|  | TD | ASD | ASD+ ADHD | ADHD | TD | ASD | ASD+ ADHD | ADHD | Main Effect:  Inclusion Status | | Interaction Effect:  Group × Inclusion | |
|  | (n=22) | (n=21) | (n=23) | (n=25) | (n=4) | (n=5) | (n=4) | (n=3) | *F* | *p* | *F* | *p* |
| **Participant characteristics** | | | | | | | | | | | | |
| Age | 23.0 (1.3) | 22.8  (0.9) | 23.1 (1.3) | 23.1 (2.0) | 25.5  (0.6) | 22.6  (0.5) | 22.8  (1.0) | 22.7  (0.6) | 1.03 | .312 | 3.67 | .015* |
| FSIQ | 118.5 (12.1) | 102.0 (19.8) | 109.2 (14.8) | 116.0 (13.2) | 111.3  (10.6) | 100.0  (12.1) | 93.3  (15.6) | 94.0  (29.3) | 7.79 | .006** | 1.11 | .348 |
| Self-rated ADHD and ASD trait | | | | | | | | | | | | |
| SDQ hyperactivity/ impulsivity | 1.7  (1.4) | 3.1  (2.0) | 7.0  (1.9) | 7.4  (1.5) | 1.8  (1.7) | 4.4  (2.5) | 6.3  (2.2) | 8.7  (1.5) | 0.95 | .333 | 1.08 | .360 |
| CAARS ADHD index | 42.1 (8.0) | 46.8 (8.5) | 58.3 (11.8) | 65.2 (7.7) | 40.3  (5.3) | 49.6  (3.9) | 68.3  (14.1) | 62.0  (15.6) | 0.57 | .453 | 1.32 | .274 |
| SRS2 total | 47.6 (6.3) | 61.8 (9.1) | 65.0 (10.3) | 62.2 (7.0) | 49.8  (7.0) | 58.0  (15.9) | 79.8  (33.8) | 57.0  (37.9) | 0.75 | .388 | 3.93 | .011* |
| Informant-rated ADHD and ASD trait | | | | | | | | | | | | |
| SDQ hyperactivity/ impulsivity | -- | 3.0  (1.6) | 7.2  (1.7) | 7.6  (1.7) | -- | 4.6  (2.4) | 6.8  (1.7) | 9.3  (1.2) | 3.19 | .078 | 1.70 | .190 |
| CAARS ADHD index | -- | 47.8 (7.3) | 66.0 (10.3) | 63.9 (10.6) | -- | 51.0  (8.1) | 74.0  (9.4) | 74.7  (1.2) | 6.34 | .014* | 0.65 | .526 |
| SRS2 total | -- | 63.4 (8.2) | 69.4 (11.5) | 57.1 (10.9) | -- | 67.2  (8.9) | 78.3  (9.0) | 65.7  (18.0) | 4.41 | .039* | 0.26 | .769 |
| Abbreviations FSIQ = full-scale IQ, CAARS = Conners Adult ADHD Rating Scale, SRS-2 = Social Responsiveness Scale version 2, SDQ = Strengths and Difficulties Questionnaires. *** *p* < .001, ** *p* < .01, * *p* < .05. | | | | | | | | | | | | |

**Table S2b. Task performance of included and excluded participants**

|  | INCLUDED | | | | EXCLUDED | | | | Statistics | | | |
| --- | --- | --- | --- | --- | --- | --- | --- | --- | --- | --- | --- | --- |
|  | TD | ASD | ASD+ ADHD | ADHD | TD | ASD | ASD+ ADHD | ADHD | Main Effect:  Inclusion Status | | Interaction Effect:  Group × Inclusion | |
|  | (n=22) | (n=21) | (n=23) | (n=25) | (n=4) | (n=5) | (n=4) | (n=3) | *F* | *p* | *F* | *p* |
| **Modified stop-signal fMRI task performance** | | | | | | | | | | | | |
| Correct Go (SD), % | 87.3  (7.55) | 87.0  (6.43) | 86.2  (7.41) | 86.4  (6.02) | 58.0  (4.88) | 60.5  (4.05) | 65.8  (14.8) | 75.5  (13.3) | 118.4 | <.0005*** | 3.74 | .014* |
| SSRT (SD), ms | 121.2  (141.0) | 135.4  (105.4) | 179.3  (97.1) | 173.0  (63.8) | 143.3 (167.1) | 272.3  (133.7) | 359.2  (569.1) | 184.7  (66.8) | 4.83 | .030* | 1.08 | .361 |
| PERTS (SD), ms | 1.0  (53.3) | 11.4  (57.8) | 18.4  (37.1) | 15.5  (55.5) | -63.1 (88.2) | -50.8 (77.8) | -41.2  (38.1) | 23.2  (34.7) | 9.12 | .003** | 1.21 | .310 |
| MRT to Go (SD), ms | 636.2  (134.8) | 586.6  (108.5) | 574.7  (102.9) | 602.3  (127.8) | 580.0  (94.3) | 670.2  (174.6) | 566.4  (82.9) | 623.0  (59.3) | 0.092 | .762 | 0.89 | .451 |
| Intrasubject RTV (SD), ms | 162.7  (46.3) | 155.6  (54.9) | 146.4  (44.8) | 151.0  (43.5) | 139.3  (45.0) | 169.1  (18.5) | 171.9  (71.3) | 210.9  (10.4) | 2.14 | .147 | 1.63 | .188 |
| **Go/No-Go task performance** | | | | | | | | | | | | |
| Prob. of inhibition (SD), % | 85.8  (11.8) | 75.8  (19.0) | 67.1  (19.1) | 66.7  (14.5) | 82.8 (8.00) | 75.0 (8.48) | 55.6 (13.0) | 69.2  (27.2) | .522 | .471 | .443 | .723 |
| Prem. responses (SD), % | 0.6  (1.0) | 1.2  (1.9) | 3.5  (8.8) | 2.3  (3.0) | 0.5  (0.8) | 0.9  (1.0) | 9.3  (8.2) | 4.9  (5.4) | 2.25 | .137 | 1.20 | .314 |
| MRT to Go (SD), ms | 301.3  (41.1) | 293.8  (34.4) | 289.5  (31.4) | 299.6  (25.3) | 282.9  (25.6) | 312.2  (52.3) | 339.2  (10.6) | 358.3  (60.2) | 8.26 | .005** | 3.30 | .023* |
| Intrasubject RTV (SD), ms | 58.3  (17.0) | 65.2  (21.6) | 69.1  (28.8) | 71.2  (19.2) | 62.0 (13.2) | 76.9 (25.6) | 136.0 (41.3) | 115.0  (56.6) | 22.7 | <.0005*** | 5.11 | .003** |
| Abbreviation MRT= mean response time, PERTS = post-error response time slowing, prem responses = premature responses, prob of inhibition = probability of inhibition, RTV = response time variability, SD = standard deviation, SSRT = stop-signal response time. *** *p* < .001, ** *p* < .01, * *p* < .05. | | | | | | | | | | | | |

# **Supplementary S3. Within-group brain activation**

1. **Successful Stop – Oddball**

On Fig. S3a, the TD group displayed activations in bilateral AI (BA13) and IFG (BA47), reaching up to right dlPFC (BA8/9) areas, bilateral supramarginal gyrus/angular gyrus (BA39/37), and STG/MTG (BA21/24), reaching into ITG in the left hemisphere, also mPFC/dACC (BA8/32), and cuneus (BA18).

| 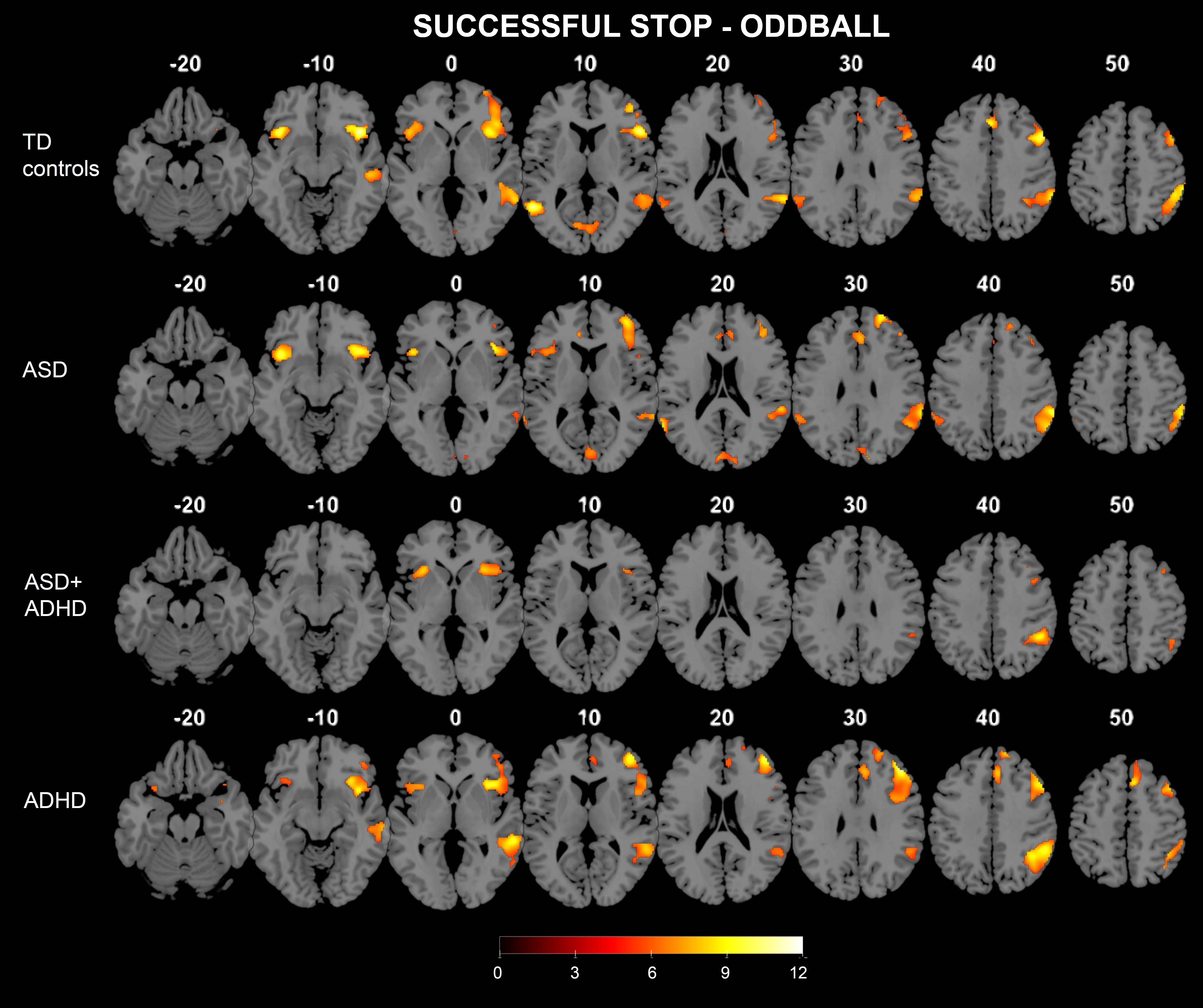 |
| --- |
| Fig. S3a. Within-group brain activations during Successful Stop relative to Oddball trials. Significant cluster of activation in the typically developing controls, ASD, ASD+ADHD and ADHD groups, obtained at a peak threshold of *p* <.001, uncorrected, and cluster extent threshold of *p* < .05 FWE_cor._ |

The ASD group showed activation at right IFG/MFG (BA46/10) and left IFG/MFG (BA44/46) reaching into AI (BA13) bilaterally, and in dACC/mPFC (BA8/32). Activation was also seen in cuneus (BA17/18), and bilaterally in IPL/supramarginal gyrus/angular gyrus (BA39/40), reaching into STG/MTG (BA21/22), more dominantly, on the right hemisphere. The ADHD group displayed activation in a large cluster comprising right AI/IFG/MFG/dlPFC/SFG (BA 13/47/45/44/46/10/9/6), dACC/mPFC, (BA24/32), left IFG/AI (BA 47/13), and right SPL/IPL/supramarginal gyrus/angular gyrus (BA7/40/39), reaching deep into right STG/MTG (BA21/22). Activations were also observed in STG/MTG (BA21/22) and premotor cortex (BA6). Finally, the ASD+ADHD group displayed the least activation relative to the other groups during successful inhibition, in IPL (BA40) and bilateral AI (BA13), reaching into right IFG (BA44/47).

| 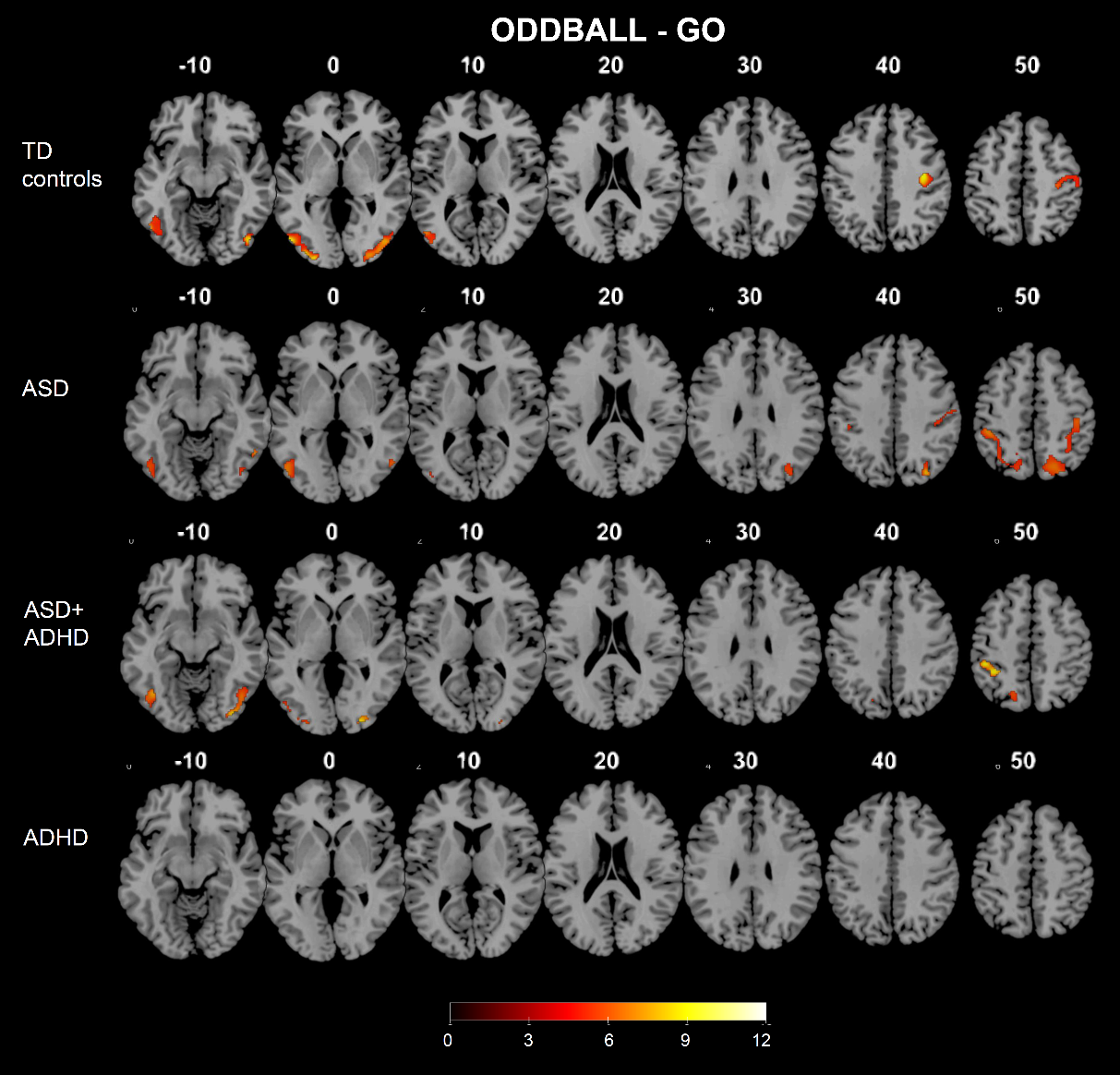 |
| --- |
| Fig. S3b. Within-group brain activations during selective attention, i.e., correct Oddball relative to Go trials. Significant cluster of activation in the typically developing controls, ASD, ASD+ADHD and ADHD groups, obtained at a peak threshold of *p* <.001, uncorrected, and cluster extent threshold of *p* < .05 FWE_cor._ No significant clusters met the threshold in the ADHD group. |

1. **Oddball – Go**

On Fig. S3b, the TD group is associated with BOLD activation in right ITG/MTG (BA37/39), and in bilateral middle/inferior occipital gyri (BA19/18). The ASD group showed extensive activation in several clusters in bilateral SPL/IPL/precuneus/ cuneus/superior occipital gyrus (BA7/19/40), extending frontally into pre- and post-CG (BA4/3/2/1) on the right hemisphere, bilaterally in inferior occipital gyrus/FFG (BA19/18), and in right PMC (BA6). Similarly, the ASD+ADHD group demonstrated brain activation in right precuneus/SPL/IPL/IPS (BA7/40), reaching frontally into right sensorimotor cortex and PMC (BA6), and ventrally into posterior MTG (BA39).

Few other clusters were observed in left SPL/IPS (BA40), and bilaterally in ITG/MTG (BA37/39) and middle and inferior occipital gyri (BA19/18). The ADHD group showed no activation meeting the threshold.

1. **Failed Stop - Successful Stop**

No significant clusters met the peak threshold of *p* <.001, uncorrected, and cluster extend threshold of *p* <.05 FWE_cor_.
